# Supplementary figures and images for: Development of Molecular Markers for Determining Continental Origin of Wood from White Oaks (Quercus L. sect. Quercus)
Source: PLoS One. 2016 Jun 28;11(6):e0158221. doi: 10.1371/journal.pone.0158221 (PMC4924829; doi:10.1371/journal.pone.0158221)

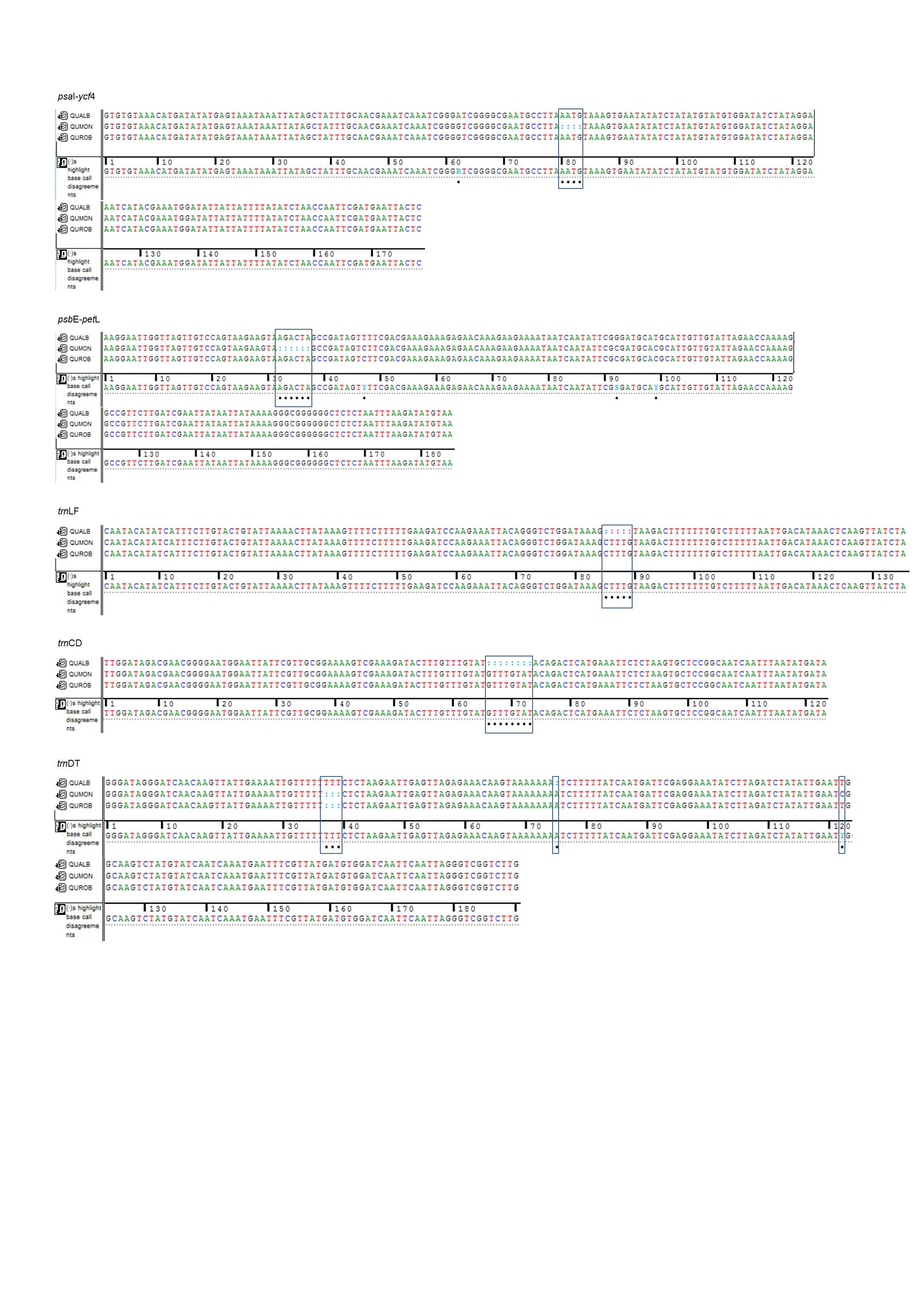

Supplement: S1 Fig — For each of the described markers an alignment of a different number of individuals (between three and 50) of three species from the three continents has been made. Thus, the sequence for each species given in the figure is a consensus sequence. Further details of the markers and the related accession numbers of single individuals are given in Table 2. QUALB: Quercus alba (USA), QUMON: Q. mongolica (Asia), QUROB: Q. robur (Europe). (TIF) [file pone.0158221.s001.tif]
